# HEalth-Related Quality of Life-Intervention in Survivors of Breast and Other Cancers Experiencing Cancer-Related Fatigue and Associated Cognitive Symptoms Using TraditionAL Chinese Medicine: The ‘HERBAL’ Trial

**DOI:** 10.1177/15347354251314514

**Published:** 2025-01-22

**Authors:** Alexandre Chan, Daniella Chan, Ding Quan Ng, Huang Fang Zheng, Quan Ming Tan, Chia Jie Tan, Jolene Hui Min Toh, Ning Yi Yap, Yi Long Toh, Yu Ke, Edmund Chun Ann Wang, Queenie Pei Ni Lim, Han Kiat Ho, Lita Chew, Tira J. Tan

**Affiliations:** 1 University of California Irvine, Irvine, CA, USA; 2 Singapore Thong Chai Medical Institution, Singapore, Singapore; 3 University of Utah, Salt Lake City, UT, USA; 4 National University of Singapore, Singapore, Singapore; 5 Subang Jaya Medical Centre, Subang Jaya, Malaysia; 6 National Cancer Centre Singapore, Singapore

**Keywords:** cancer-related fatigue, traditional Chinese medicine, Xiang Bei Yang Rong Tang, integrative oncology, cancer-related cognitive impairment, quality of life

## Abstract

**Introduction:**

As pharmacological strategies remain limited for relieving fatigue and associated cognitive symptoms, integrative modalities such as traditional Chinese medicine (TCM) could be explored as therapeutic strategies in cancer survivors. Here, we evaluate and report the efficacy and safety of a TCM concoction, modified Xiang Bei Yang Rong Tang (XBYRT), on quality of life (QOL), cancer-related fatigue (CRF), and cognitive symptoms, compared to placebo.

**Methods:**

In a single-centered, randomized, double-blinded, placebo-controlled pilot trial conducted from 2019 to 2022, fatigued cancer survivors ≥21 years old were recruited to receive the XBYRT intervention or placebo (5% diluted) once daily for the duration of 8 weeks. Patient-reported outcomes for QOL, CRF, cognition, blood samples for biomarker testing, and adverse events were collected at baseline (T0), 4 weeks (T1), 8 weeks (T2), and 10 weeks (T3) after baseline. Linear regression was performed to evaluate differences between groups at T2 and T3.

**Results:**

A total of 1502 patients were screened, with 672 patients considered eligible. Of the eligible, 15 XBYRT and 13 placebo subjects with similar mean ages (58.5 vs 58.4) were recruited. Both groups were predominantly Chinese (93% vs 62%), breast cancer patients (87% vs 62%), and diagnosed with stage 2 cancer (60% vs 46%). Although no significant difference was found in QOL between groups, the XBYRT group exhibited improved emotional fatigue at T3 (*P* = .045) and higher BDNF levels at T2 (*P* = .047) and T3 (*P* = .029). After baseline adjustment, XBYRT was associated with better perceived cognitive impairment at T2 (*P* = .011) and T3 (*P* = .017), as well as overall perceived cognitive function at T3 (*P* = .028). XBYRT is well tolerated, with grade 3 adverse events reported in three XBYRT (20%) and two placebo (15%) subjects.

**Conclusion:**

In this pilot study, XBYRT as an integrative therapy is safe and generates encouraging improvements in cognitive and fatigue symptoms. Difficulties with recruitment limited the generalizability of trial findings, thus findings should be verified through a larger, multi-centered trial.

## Introduction

Cancer patients are surviving longer due to advances in diagnosis and treatment. However, they are now faced with wide-ranging debilitating toxicities as a consequence of these treatments, some lasting years after treatment cessation. Cancer-related fatigue (CRF), occurring in up to 85% of cancer survivors,^1^ is characterized by a distressing, persistent, subjective sense of physical, emotional, and/or cognitive tiredness that negatively impacts quality of life (QOL). The lasting impact of CRF was evident in up to 52% of cancer survivors 3-years post diagnosis,^1^ and 42% of working cancer survivors.^2^ Despite these consequences, there is limited understanding of the underlying pathogenesis and pharmacological treatments remain investigational with limited efficacy.

Consequently, many cancer survivors seek complementary and alternative medicine to address general health and symptoms that persist despite conventional treatments, of which traditional Chinese medicine (TCM) is commonly used in Asia.^3-5^ We have designed a concoction in collaboration with certified TCM practitioners, namely Xiang Bei Yang Rong Tang (香贝养荣汤, XBYRT), which contains 15 herbal components (Table 1) aimed at improving patients’ QOL and reducing CRF and associated symptoms. Notably, the concoction contains *Radix Astragali seu Hedysari and Rhizoma Atractylodis*,^6^ which are known to improve fatigue, and C*odonopsis Pilosula*,^7^ which is frequently used to ameliorate chronic fatigue syndrome. Other individual herbal components such as *Fructus Lycii*, *Fructus Ligustri Lucidi*, and *Fructus Alpinia*, have demonstrated neuroprotective effects by reducing the accumulation of reactive oxygen species, a known contributor to the pathogenesis of neurogenerative diseases.^8-10^ Additionally, extracts of *Radix Polygalae*^11^ show neuroprotective effects against oxidative stress and apoptosis, while also improving nerve growth, neuronal plasticity, neurotransmitter reuptake, and neurogenesis, likely by increasing brain-derived neurotrophic factor (BDNF) expression via regulation of the cyclic AMP-responsive element-binding protein (CREB)-dependent pathway.^12^ In our in-vitro toxicology studies, XBYRT has a favorable toxicity profile and drug interaction profile.^13^

**Table 1.** Formula and Components of the Modified Xiang Bei Yang Rong Tang Decoction.

Chinese name	Chinese name (Pinyin)	Scientific name	Dosage (g)	Purported effect
黄芪	Huang Qi	*Radix Astragali seu Hedysari*	15	Augments Qi and raises Yang, augments defensive-Qi, consolidates the superficies, promotes drainage of pus and healing, facilitates water movement and reduces swelling
党参	Dang Shen	*Radix Codonopsis pilosulae*	15	Tonifies the middle-jiao, augments Qi and generates fluids and blood
白术	Bai zhu	*Rhizoma Atractylodis macrocephalae*	12	Augments Qi, strengthens the spleen, dries dampness, promotes diuresis, stops sweating
茯苓	Fu Ling	*Poria*	15	Drains water, dissipates dampness, strengthens the spleen and calms the mind
白芍	Bai shao	*Radix Paeoniae alba*	15	Nourishes blood, retains Yin, soothes the liver and relieves pain, and stops excessive perspiration
枸杞子	Guo Qi zi	*Fructus Lycii*	12	Nourishes the liver and kidney, clears the eyes and moistens the lung
女贞子	Nü Zhen Zi	*Fructus Ligustri lucidi*	12	Tonifies the liver and kidney, cools heat and clears the eye
车前子	Che Qian Zi	*Plantago asiatica*	12	Induces diuresis, drain dampness, improve vision and resolve phlegm
鸡内金	Ji Nei Jin	*Endothelium Corneum gigeriae Galli*	10	Promotes digestion and invigorate spleen, arrest seminal emission and relieve enuresis
生麦芽	Shen Mai Ya	*Hordeum vulgare L.*	15	Promotes digestion and invigorate spleen, stop lactation and release distension
益智仁	Yi Shin Ren	*Fructus Alpinia oxyphylla*	10	Tonifies kidney yang, secure essence and reduce urination, warm spleen yang, improve appetite and reduce salivation
香附	Xiang Fu	*Rhizoma Cyperi*	10	Unblocks the liver and regulates Qi, regulates menstruation and stops pain
远志	Yuan Zhi	*Radix Polygalae*	10	Stabilizes the heart and calms the mind, dissolves phlegm and opens orifices, and reduces abscesses and swelling
浙贝母	Zhe bei mu	*Bulbus Fritillariae thunbergii*	10	Clears heat and resolves phlegm, disperses masses/abnormal growth and promotes the healing of carbuncles
土茯苓	Tu Fu Ling	*Smilax glabra Roxb*	15	Removes toxicity, excrete dampness and ease joint movement

To evaluate the efficacy of XBYRT in cancer survivors, we have conducted a randomized controlled pilot trial on patients’ QOL and CRF, with the goal to evaluate its role as an integrative oncology modality. Additionally, to evaluate its impact on physiological function, symptoms and safety, we also aim to compare the perceived cognitive function, plasma biomarkers and safety outcomes to comprehensively quantify the impact of XBYRT on cancer survivors.

## Methods

This manuscript was prepared following the CONSORT 2010 statement (Supplemental Table S1).

### Trial Design

The study was a randomized, double-blinded, placebo-controlled, parallel trial, conducted from October 2019 to October 2022 (ClinicalTrials.gov: NCT04104113, September 26, 2019). The study received SingHealth Centralized Institutional Review Board (IRB) approval (CIRB No.: 2019/2135) and the protocol was previously published.^14^

### Participants

We recruited cancer survivors through oncologist referral at the National Cancer Centre Singapore (NCCS). Eligible participants were ≥21 years old, reported a fatigue screen score of ≥4 in the past 7 days (0, no fatigue; 10, worst fatigue) as recommended by ASCO cancer-related fatigue guidelines,^15^ had completed surgery, chemotherapy, or radiotherapy and were not planned on receiving adjuvant therapy during the study period, except for aromatase inhibitors or ovarian suppression for breast cancer survivors. Patients with metastases, cancer recurrence, untreated fatigue-causing co-morbidities, on fatigue-inducing medications, taking warfarin, receiving or planning to receive TCM treatment, or breastfeeding or intending to conceive were excluded. Certified TCM physicians also screened the potential participants to ensure patient eligibility to receive the concoction based on the TCM syndrome differentiation. According to TCM principles, satisfaction of the TCM syndrome differentiation involves the deficiency of qi and blood by experiencing either: (1) two major symptoms with typical pulse and tongue conditions or (2) one major symptom and two possible symptoms with pulse and tongue conditions (Supplemental Table S2). The National Standard in People’s Republic of China and the Clinical Practice Guidelines of Chinese Medicine in Oncology currently endorses this method of condition identification.^16^

### Intervention

Study participants were randomized to either the XBYRT or placebo arms, and participants were given investigational products prepared as granules designed to be dissolved in hot water for once daily consumption for 8 weeks. In the XBYRT arm, participants received a 24 g daily dosage, selected based on the safety and efficacy guidelines from the *Pharmacopoeia of the People’s Republic of China and Chinese Materia Medica Textbook for Higher Education*^17,18^ (Table 1). The placebo granules comprised 5% of the herbal components, 95% maltodextrin, 0.002% denatonium benzoate as bitterant and colorant to ensure that its taste and smell is similar to XBYRT treatment while minimizing therapeutic effect. Participants were reminded to take their daily dosages and record missed dosages. The granules were manufactured by Kinhong Pte Ltd., Singapore, a Good Manufacturing Practices-certified manufacturer.

To advise on safe concomitant administration with medications, we had previously evaluated the potential interaction of XBYRT with the activities of CYP3A4 and CYP2D6 and found that the herbal components did not inhibit the CYP enzymes. Further, resulting liver cell viability demonstrated that XBYRT is not likely to cause hepatotoxicity.^13^

### Assessments

Before treatment initiation, participants’ demographics, cancer diagnosis, medical history, and concomitant medications were recorded. Questionnaires, blood draws and safety monitoring were completed at four time points: baseline (T0) and 4 weeks (T1), 8 weeks (T2), and 10 weeks (T3) after baseline. Pulse conditions were also assessed at four time points to ensure patient safety. Due to the COVID-19 pandemic, subsequent pulse assessments were conducted through teleconsultations (phone or video call) or to be omitted. Based on Singapore’s TCM Practitioners Board guidelines, while the first visit must be in person, follow ups can be virtual.

#### Quality of life (QOL)

Health-related QOL was measured using the global health status (GHS) domain of the EORTC Core Quality of Life questionnaire (EORTC QLQ-C30) questionnaire. A higher score indicated higher QOL.

#### Cancer-related fatigue (CRF)

The Multidimensional Fatigue Symptom Inventory-Short Form (MFSI-SF) measures CRF across five subscales: general, physical, emotional fatigue, and mental fatigue, and vigor. Except for the vigor subscale, higher scores in each domain represented more fatigue. An overall score was obtained by combining subscale scores (subtracting vigor scores), with higher scores indicating worse fatigue symptoms. We have previously performed a study to evaluate the psychometric properties and measurement equivalence of the English and Chinese versions of MFSI-SF in breast cancer and lymphoma patients in Singapore.^19^

#### Perceived cognition

Perceived cognitive function was assessed using the FACT-Cog v3, which produced four subscales’ scores: perceived cognitive impairment (CogPCI), perceived cognitive abilities (CogPCA), impact of perceived cognitive impairment on QOL (CogQOL), and comment from others on cognitive function (CogOTH). Totaling the subscale scores generated the total score, with better perceived cognition indicated by higher scores. We have previously evaluated the psychometric property and measurement equivalence of the English and Chinese versions of FACT-Cog v3 among breast cancer patients in Singapore.^20^

#### Safety monitoring

Safety was assessed through patient reports and blood tests evaluating toxicities to organ functions (eg, renal and liver function tests, full blood count, and electrolyte level). Adverse events (AEs) were recorded and graded by research nurses using the Common Terminology Criteria for Adverse Events (CTCAE) version 5.

#### Blood plasma processing and storage

A 9 mL blood sample was collected in ethylenediaminetetraacetic acid tubes. Subsequently, it was then centrifuged for 10 minutes at 1069 × *g* at 4ºC. Aliquots of plasma and buffy coat were stored at −80ºC until analysis.

#### Inflammatory cytokines

Plasma levels of interleukin (IL)-2, IL-4, IL-6, IL-8, IL-10, granulocyte-macrophage colony-stimulating factor (GM-CSF), interferon (IFN)-γ, and tumor necrosis factor (TNF)-α, were quantified using 50 µL of each sample with a highly sensitive multiplex immunoassay (Bioplex Human Cytokine 9-Plex Panel, Bio-Rad, USA).

#### Plasma brain-derived neurotrophic factor (BDNF) levels

We quantified BDNF levels with an enzyme-linked immunosorbent assay (ELISA) kit (Biosensis BEK-2211-1P/2P, Australia) using 100 µL of sample diluted 100-fold.

#### BDNF Val66Met genotyping (rs6265)

Genomic DNA was isolated from the buffy coat with QIAmp DNA Blood Mini Kit (Qiagen, Germany) and was polymerase chain reaction (PCR) amplified with forward primers (5′-GGACTCTGGAGAGCGTGAA-3′) and reverse primers (5′-CGTGTACAAGTCTGCGTCCT-3′). PCR products genotyping was completed by automated Sanger sequencing with a 3730xl DNA Analyzer (Applied Biosystems, USA).

### Outcomes

Our primary endpoint was the difference of QOL scores between treatment and control groups, hence the primary analysis involved comparing the T2 and T3 GHS scores between the treatment and control groups. Secondary analyses included comparing MFSI-SF and FACT-Cog total and subscale scores, plasma BDNF and cytokine levels, at T2 and T3, as well as prevalence of AEs between the two arms. Exploratory outcomes included the association of the biomarkers with patient-reported outcomes (PROs).

### Sample Size

To ensure an adequate sample size to inform a future phase III trial design, we aimed to recruit 80 subjects (40 per arm) as recommended by Teare et al.^21^ after accounting for a 10% dropout rate.

### Randomization

Block randomization with a block size of 10 was performed by a third-party clinical trial service provider using the sealed envelope method. The physicians, trial pharmacists, study team, and study participants were blinded to the block size, randomization sequence and treatment assignment.

### Statistical Methods

Baseline characteristics were descriptively summarized using counts and percentages for categorical variables, and means and standard deviations (SD) for continuous variables. Differences in all outcomes were tested at T2 and T3 using linear regression with or without baseline adjustment. Further sensitivity analysis was performed for significant outcomes by adjusting for rs6265 genotypes which are known to influence patient outcomes.^22^ All analyses were two-tailed, tested at 5% significance level, and conducted on R version 4.3.2.

## Results

### Participants

**Figure 1.**
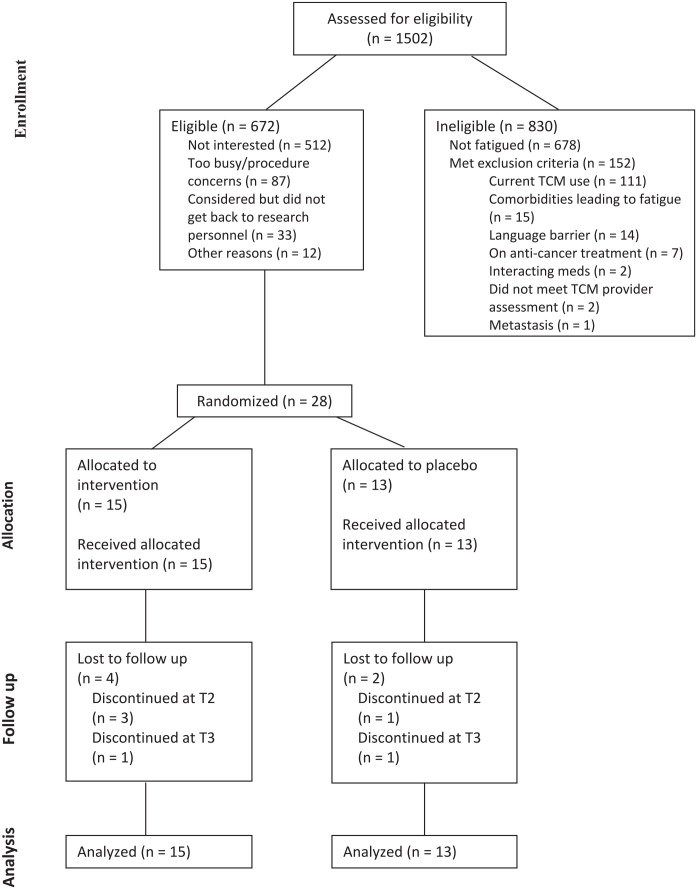
Subject CONSORT diagram.

A total of 1502 cancer survivors were screened for eligibility between October 2019 to October 2022, of which 830 were ineligible as they were either not fatigued (n = 678) or met exclusion criteria (n = 152). Of the 672 that were eligible, 28 survivors entered the trial. The rest of the survivors did not enroll for the following reasons: not interested (n = 512), too busy or had procedure concerns (n = 87), were considering participation but did not get back to research personnel (n = 33), and other reasons (n = 12; Figure 1).

Fifteen XBYRT and 13 placebo subjects with comparable mean ages (58.5 vs 58.4) were recruited (Table 2). Both groups mainly consisted of Chinese (93% vs 62%), breast cancer patients (87% vs 62%) and stage II cancer diagnoses (60% vs 46%). Surgery, chemotherapy, and radiotherapy were the most common treatment modalities in both groups. There was a lower proportion of rs6265 Met carriers among the XBYRT arm compared to placebo (47% vs 77%). We observed no difference between the two groups for all PROs and continuous biomarkers at baseline (all *P* > .05, Supplemental Tables S3 and S5). Recruitment was hindered as the trial was conducted during the COVID-19 pandemic starting in 2020, resulting in delays, and ultimately had to close prior to achieving the planned sample size due to funding issues.

**Table 2.** Baseline Characteristics of XBYRT and Placebo Groups.

Variables	XBYRT group (N = 15)	Placebo group (N = 13)
*Age at survey*		
Mean (SD)	58.5 (6.3)	58.4 (10.0)
*Sex*, n (%)		
Male	0 (0.0)	1 (7.7)
Female	15 (100.0)	12 (92.3)
*Race/ethnicity*, n (%)		
Chinese	14 (93.3)	8 (61.5)
Indian	0 (0.0)	2 (15.4)
Malay	0 (0.0)	1 (7.7)
Other	1 (6.7)	2 (15.4)
*BMI*		
Mean (SD)	25.4 (4.5)	25.8 (4.9)
*Menopausal state*, n (%)		
Pre-menopausal	1 (6.7)	2 (15.4)
Post-menopausal	14 (93.3)	10 (76.9)
Male	0 (0.0)	1 (7.7)
*Cancer type*, n (%)		
Breast	13 (86.7)	8 (61.5)
Lymphoma	0 (0.0)	2 (15.4)
Endometrial	1 (6.7)	0 (0.0)
Pancreatic	0 (0.0)	1 (7.7)
Ovarian	0 (0.0)	1 (7.7)
Lung	1 (6.7)	0 (0.0)
Uterine	0 (0.0)	1 (7.7)
*Cancer stage*, n (%)		
I	3 (20.0)	4 (30.8)
II	9 (60.0)	6 (46.2)
III	3 (20.0)	3 (23.1)
*Education level*, n (%)		
Primary	1 (6.7)	0 (0.0)
Secondary	6 (40.0)	6 (46.2)
Pre-university	1 (6.7)	1 (7.7)
Graduate/postgraduate	7 (46.7)	6 (46.2)
*Employed*, n (%)		
Yes	9 (60.0)	5 (38.5)
No	6 (40.0)	8 (61.5)
*ECOG*, n (%)		
0	15 (100.0)	11 (84.6)
1	0 (0.0)	2 (15.4)
*Treatment received*, n (%)		
Radiotherapy	10 (66.7)	7 (53.9)
Chemotherapy	9 (60.0)	13 (100.0)
Targeted therapy	3 (20.0)	4 (30.8)
Hormonal therapy (completed)	4 (26.7)	1 (7.7)
Hormonal therapy (ongoing)	4 (26.7)	4 (30.8)
Surgery	15 (100.0)	11 (84.6)
*Comorbidities*, n (%)		
Hyperlipidemia	3 (20.0)	3 (23.1)
Hypertension	2 (13.3)	3 (23.1)
Insomnia	2 (13.3)	2 (15.4)
Hyperthyroidism	1 (6.7)	0 (0.0)
Hypothyroidism	0 (0.0)	3 (23.1)
Diabetes	1 (6.7)	1 (7.7)
Depression	0 (0.0)	2 (15.4)
*EORTC QLQ-C30*, mean (SD)		
GHS score	53.3 (14.7)	51.3 (21.5)
*MFSI-SF*, mean (SD)		
General scale	12.4 (5.2)	11.2 (5.8)
Physical scale	10.2 (4.7)	8.8 (4.5)
Emotional scale	5.7 (4.5)	7.0 (3.9)
Mental scale	7.2 (5.1)	7.0 (3.9)
Vigor scale	10.0 (4.1)	10.1 (4.5)
Total score	25.5 (19.3)	23.9 (18.2)
*FACT-Cog*, mean (SD)		
Cog-PCI	50.4 (16.2)	52.9 (14.8)
Cog-QOL	10.2 (4.5)	8.8 (3.8)
Cog-Oth	14.5 (2.0)	14.8 (2.0)
Cog-PCA	16.7 (5.4)	15.8 (6.9)
Total score	94.4 (23.0)	92.2 (24.3)
*BDNF SNP rs6265*, n (%)		
Val/Val	7 (46.7)	3 (23.1)
Met carriers (Val/Met, Met/Met)	7 (46.7)	10 (76.9)
Unknown	1 (6.7)	0 (0.0)
*Plasma biomarkers*, mean (SD)		
BDNF (pg/mL)	5725.4 (7513.7)	2722.5 (4299.5)
IL-2 (pg/mL)	0.8 (0.9)	0.3 (0.4)
IL-4 (pg/mL)	0.1 (0.3)	0.0 (0.0)
IL-6, (pg/mL)	0.5 (0.3)	0.8 (1.2)
IL-8 (pg/mL)	3.05 (1.37)	2.95 (1.42)
IL-10 (pg/mL)	1.0 (0.5)	1.0 (0.4)
GMCSF (pg/mL)	0.0 (0.1)	0.0 (0.0)
IFNg (pg/mL)	0.9 (0.5)	0.9 (0.5)
TNFα (pg/mL)	10.8 (5.3)	12.9 (5.7)

Abbreviations: BDNF, brain-derived neurotrophic factor; BMI, body mass index; ECOG, Eastern Cooperative Oncology Group; EORTC QLQ-C30, European Organization for Research and Treatment of Cancer Quality of Life Questionnaire—C30; FACT-Cog, fatigue assessment of cancer therapy—cognitive function; GHS, global health score; GMCSF, granulocyte-macrophage colony-stimulating factor; IFNg, interferon gamma; IL, interleukin; MFSI-SF, multidimensional fatigue symptom inventory—short form; PCA, perceived cognitive abilities; PCI, perceived cognitive impairments; Q, quartile; QOL, quality of life; SD, standard deviation; SNP, single nucleotide polymorphism; T0, timepoint 0; T1, timepoint 1; T2, timepoint 2; T3, timepoint 3; TNFα, tumor necrosis factor alpha; XBYRT, Xiang Bei Yang Rong Tang.

### QOL, CRF, and Perceived Cognition

**Figure 2.**
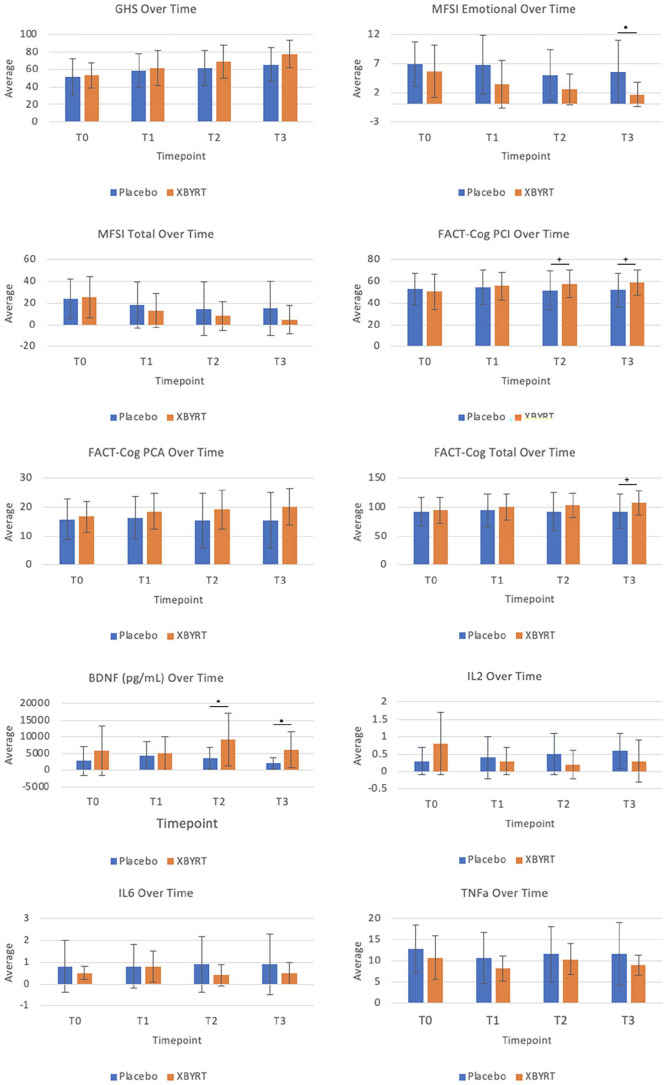
Assessment and biomarker measurements across all timepoints between the XBYRT and placebo groups. *P < .05 without baseline adjustment. ^+^*P* < .05 with baseline adjustment.

Descriptively, a widening gap between the XBYRT and placebo groups was observed for all PRO (Figure 2 and Supplemental Table S3) over the course of the trial. However, we found no difference in GHS scores (primary endpoint) at T2 and T3 between the groups (Supplemental Tables S3 and S4).

Regarding CRF, the XBYRT arm demonstrated better emotional fatigue symptoms at T3 (*P* = .045, β = −3.82, 95% CI = −7.53 to −0.10) (Supplemental Table S3). Statistical significance was not reached at other timepoints nor other MFSI-SF subscales.

Regarding perceived cognition, no difference was detected in the FACT-Cog subscales across all time points between the two groups. However, after adjusting for baseline, the XBYRT group exhibited better perceived cognitive function, represented by CogPCI, at T2 (*P* = .011, β = 8.27, 95% CI = 2.07 to 14.5) and T3 (*P* = .017, β = 8.97, 95% CI = 1.77 to 16.2) (Supplemental Table S4). Moreover, a better total FACT-Cog score at T3 (*P* = .028, β = 15.4, 95% CI = 1.86 to 29.0) was exhibited amongst the XBYRT group.

### Biomarkers

The XBYRT group demonstrated significantly higher BDNF levels (in pg/mL) at T2 (*P* = .047, β = 5598, 95% CI = 94 to 11 103) and T3 (*P* = .029, β = 4010, 95% CI = 452 to 7570) (Supplemental Table S5), although statistical significance was not achieved after baseline adjustment. No statistical significance was found for other biomarkers (Supplemental Table S5 and S6).

### Sensitivity Analysis: Adjusting for rs6265 Genotypes

In a sensitivity analysis adjusting for rs6265 genotypes (Val/Val vs Met carriers), FACT-Cog CogPCI and total scores at T3 (after baseline adjustment), and BDNF levels at T2 and T3 remained significantly higher among XBYRT (*P* < .05, data not reported). In these models, Met carriers scored significantly lower in FACT-Cog total scores at T3 (*P* = .049, β = −14.9, 95% CI = −29.7 to −0.04).

### Safety

In the XBYRT arm, grade 3 adverse events were reported in three patients (20%): one experienced insomnia at T1; the second patient experienced insomnia and headache at T1; the third experienced headache at T2. In the placebo arm, two subjects (15%) experienced grade 3 adverse events: one experienced constipation at T1; the second patient experienced insomnia (T1 and T2), dry mouth (T2 and T3), and flushing (T2 and T3). Safety findings are summarized in Supplemental Table S7.

### Exploratory Analysis: BDNF-PRO Spearman Correlation Analysis

**Figure 3.**
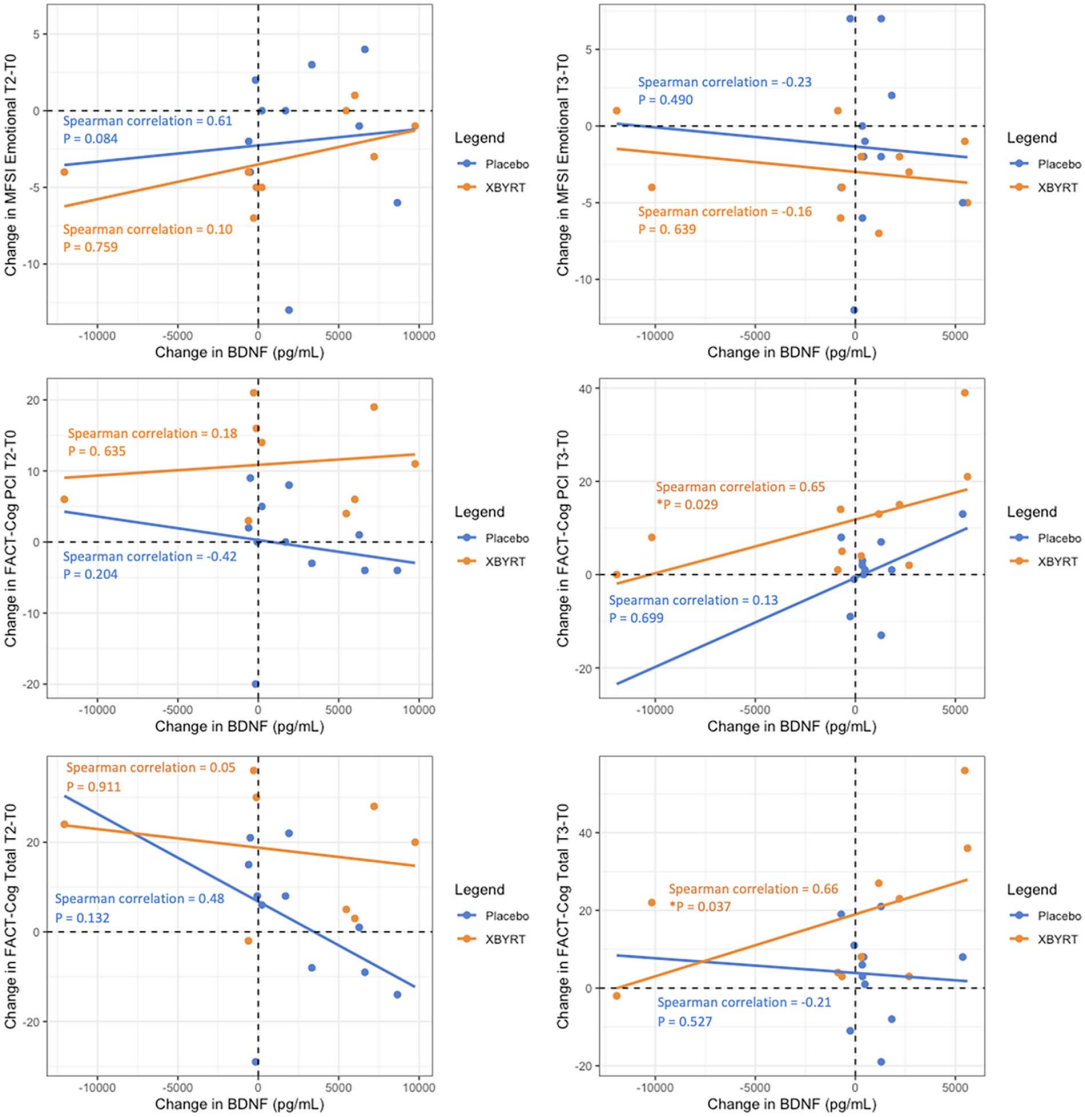
Spearman correlation coefficients for differences between T2 and baseline and T3 and baseline for PROs. *P < .05.

Because XBYRT treatment appears to influence emotional fatigue, perceived cognition and BDNF levels, an exploratory Spearman correlation analysis was conducted to explore the relationship between BDNF and the various PROs. Within the XBYRT group, increase in BDNF levels is significantly associated with the increase in FACT-Cog CogPCI and total scores, from T0 to T3 (*P* < .05, Figure 3). No other statistically significant relationships were observed.

## Discussion

In this randomized controlled pilot trial of herbal medicine for CRF, we found an improvement of fatigue and cognitive symptoms among those treated with XBYRT compared to placebo. We observed a significant sustained improvement after treatment for both emotional fatigue and perceived cognition. Our analysis of plasma biomarkers reported significant upregulation in BDNF levels after 8 weeks of XBYRT, although this increase was not sustained after treatment cessation. This increase in BDNF levels was associated with improved cognitive function after cessation of XBYRT therapy. We did not observe any significant safety concerns with the concoction. Taken together, we conclude that the XBYRT treatment demonstrated encouraging efficacy and safety data that warrant further investigation as a potential clinically feasible treatment for CRF and cancer-related cognitive impairment (CRCI).

According to theories of TCM, CRF is characterized both by a deficiency in qi, the body’s vital energy, and in blood.^23,24^ Chemotherapy and radiotherapy can result in impairment and consumption of Qi and blood, as these anti-cancer treatments suppress bone marrow, are cytotoxic, and result in fatigue, weakness, lack of energy or decline in physical functioning, causing the body to be in a deficiency state. It has been found that a qi deficiency correlated with CRF and worse QOL among cancer patients.^5^ Although there was no impact in overall QOL in our study, we observed an improvement in emotional fatigue and perceived cognitive function in the intervention arm. These clinical outcomes are consistent with the therapeutic intent for individual ingredients of XBYRT, which includes *Radix Astragali seu Hedysari and Rhizoma Atractylodis macrocephalae.*^6^ In a network pharmacology analysis, it was suggested that the mechanism of action of *Radix Astragali seu Hedysari* and *Rhizoma Atractylodis macrocephalae* on CRF mainly involved compounds, such as quercetin, kaempferol and luteolin, acting through multiple targets, such as protein kinase AKT1, tumor necrosis factor (TNF), and IL-6. Other individual herbal components such as *Fructus Lycii*, *Fructus Ligustri lucidi*, and *Fructus Alpinia oxyphylla*, have demonstrated neuroprotective effects. In the literature, *Fructus Ligustri lucidi* and *Fructus Alpinia oxyphylla* contain ethyl acetate extracts and sesquiterpenoids, respectively, which have shown to reduce the accumulation of reactive oxygen species, a known contributor to the pathogenesis of neurogenerative diseases.^9,10^ However, we did not observe a statistically significant reduction in TNFα and IL-6 biomarkers in the XBYRT arm which may be attributed to the small study sample size. Moreover, studies have shown that oligosaccharide esters from *Radix Polygalae*, tenuifoliside, and 3,6′-disinapoylsucrose (DISS), increase BDNF expression via regulation of the CREB-dependent pathway.^12^ These may explain the improvement of better perceived cognitive function and higher BDNF levels post XBYRT.

Interestingly, Met carriers of *BDNF* rs6265 single nucleotide polymorphism (SNP) were not as responsive to the treatment compared to participants who are homozygous for the wildtype (Val) allele. This illustrates the potential utility of the SNP to screen for Val/Val cancer survivors who may benefit most from receiving XBYRT. Correspondingly, these patients were also most in need of such interventions as they were predisposed with a higher risk of declines in cognition and BDNF downregulation after cancer treatment based on our previous longitudinal studies of breast and young adult cancer survivors from Singapore.^22,25-27^ Findings in the literature, however, had not been consistent across studies conducted in other countries.^28^ Inconsistent findings were similarly noted for rs6265 and CRF, with Met alleles showing a protective effect against fatigue symptoms in male^29^ cancer survivors but posing a risk in females.^30^ Differences in the relationship between rs6265 and CRF could be a function of gender, ethnicity, culture, and cancer type and should be investigated in a well-powered study.

There have been previous interventional studies which have evaluated various TCM concoctions containing multiple herbs in ameliorating CRF. For a study performed in Korea, 40 patients with CRF were randomized into either the control or intervention (Bu-Zhong-Yi-Qi-Tang [BZYQT]). The main ingredients of this decoction include *Radix Astragali*, *Radix* Ginseng, and *Atractylodis lanceae rhizome*. Statistically significant improvement in fatigue (*P* < .05) was found in patients receiving BZYQT for 2 weeks.^31^ However, the short duration of the intervention poses a study limitation. In a single-arm study with the intent to correct qi deficiency, the efficacy of Ren Shen Yangrong Tang (RSYRT) was investigated among 33 cancer survivors who reported moderate to severe fatigue.^32^ The formula includes ingredients such as *Radix Codonopsis*, *Rhizoma atractylodis macrocephalae* and *Radix rehmannia*. Patients were found to have a significant decrease fatigue severity after receiving the intervention for the course of 6 weeks. Moreover, in addition to all the patients experiencing subjective improvement within 4 weeks of the intervention, there was a statistically significant decrease in fatigue severity score using the MD Anderson Symptom Inventory-C (*P* < .001). However, these findings are likely biased by the lack of adequate blinding and control. In another double-blinded, placebo-controlled, randomized study of colon and breast cancer patients (n = 120), Chinese herbal medicine was given to manage nausea but not hematologic toxicity.^33^ In this study, between baseline and each chemotherapy cycle, the score change for each EORTC-QLQ-C30 domain was compared. However, no statistically significant difference was found between the placebo and TCM groups.

Considering the shortcomings of these prior studies, our trial has taken multiple approaches, including a placebo-controlled randomized design to evaluate the efficacy of our concoction. The adoption of this trial design to generate evidence demonstrating efficacy of XBYRT was a major strength of our study. To establish the role of TCM as a viable option for supportive care in cancer, the same standards imposed on pharmacological trials should also apply. Our study incorporated several unique and essential features that future trials should consider when evaluating herbal concoctions. Firstly, given the variation in composition of herbal concoctions, we have previously conducted toxicology studies of XBYRT to identify potential pharmacokinetic interactions and ensure patients who were on medications at risk of interactions with XBYRT were excluded from the study.^13^ Additionally, an active placebo at 5% strength of XBYRT was used. As the taste of herbal concoctions is widely known to be strong and typically bitter, the use of an active placebo was critical to prevent participants from recognizing that they were on the placebo arm. Maintaining blinding of participants is especially crucial given the use of patient-reported tools in our study.

However, we encountered significant challenges with recruitment, achieving 35% of the recruitment target, due to a variety of reasons including COVID-19. Nearly half (45%) of the approached patients were deemed ineligible due to low or absent fatigue levels, 34% cited a lack of interest to participate due to concerns with TCM usage, and 7% were excluded due to current TCM use. To address similar recruitment challenges in future trials of TCM, the expected duration of recruitment should be adjusted based on the prevailing acceptability of TCM within the target population. Moreover, aligning trial recruitment with ongoing symptom monitoring activities presents a promising strategy to ensure that the trial can be suitably offered to patients with reported symptom(s) of interest, such as fatigue.

## Conclusion

This randomized controlled pilot trial found XBYRT as a potentially safe integrative therapy that produced encouraging improvements in cognitive and fatigue symptoms. It is, however, necessary to keep in mind the potential challenge of applying standardized TCM decoctions to patients when designing studies in the future, as individualized decoctions may be more practical or optimal for treatment. To verify our findings, a larger, multi-centered trial will inform whether XBYRT is an appropriate intervention to manage and improve symptoms in cancer patients and survivors.

## Supplemental Material

sj-docx-1-ict-10.1177_15347354251314514 – Supplemental material for HEalth-Related Quality of Life-Intervention in Survivors of Breast and Other Cancers Experiencing Cancer-Related Fatigue and Associated Cognitive Symptoms Using TraditionAL Chinese Medicine: The ‘HERBAL’ Trial Supplemental material, sj-docx-1-ict-10.1177_15347354251314514 for HEalth-Related Quality of Life-Intervention in Survivors of Breast and Other Cancers Experiencing Cancer-Related Fatigue and Associated Cognitive Symptoms Using TraditionAL Chinese Medicine: The ‘HERBAL’ Trial by Alexandre Chan, Daniella Chan, Ding Quan Ng, Huang Fang Zheng, Quan Ming Tan, Chia Jie Tan, Jolene Hui Min Toh, Ning Yi Yap, Yi Long Toh, Yu Ke, Edmund Chun Ann Wang, Queenie Pei Ni Lim, Han Kiat Ho, Lita Chew and Tira J. Tan in Integrative Cancer Therapies
